# Asthma-Related Knowledge and Practices among Mothers of Asthmatic Children: A Latent Class Analysis

**DOI:** 10.3390/ijerph19052539

**Published:** 2022-02-22

**Authors:** Salvatore Fasola, Velia Malizia, Giuliana Ferrante, Amelia Licari, Laura Montalbano, Giovanna Cilluffo, Stefania La Grutta

**Affiliations:** 1 Institute of Translational Pharmacology, National Research Council, 90146 Palermo, Italy; velia.malizia@ift.cnr.it (V.M.); dr.montalbanolaura@gmail.com (L.M.); 2 Department of Surgical Sciences, Dentistry, Gynecology and Pediatrics, Pediatric Division, University of Verona, 37134 Verona, Italy; giuliana.ferrante@univr.it; 3 Department of Pediatrics, Fondazione IRCCS Policlinico San Matteo, University of Pavia, 27100 Pavia, Italy; amelia.licari@unipv.it; 4 Department of Earth and Marine Sciences, University of Palermo, 90123 Palermo, Italy; giovanna.cilluffo@unipa.it

**Keywords:** asthma, children, knowledge, latent profiles, disease management, mothers, practices

## Abstract

Mothers’ knowledge about childhood asthma influences management practices and disease control, but validating knowledge/practice questionnaires is difficult due to the lack of a gold standard. We hypothesized that Latent Class Analysis (LCA) could help identify underlying mother profiles with similar knowledge/practices. A total of 438 mothers of asthmatic children answered a knowledge/practice questionnaire. Using answers to the knowledge/practice questionnaire as manifest variables, LCA identified two classes: Class 1, “poor knowledge” (33%); Class 2, “good knowledge” (67%). Classification accuracy was 0.96. Mothers in Class 2 were more likely to be aware of asthma-worsening factors and indicators of attacks. Mothers in Class 1 were more likely to prevent exposure to tobacco smoke (91.1% vs. 78.8%, *p* = 0.005). For attacks, mothers in Class 2 were more likely to go to the emergency department and follow the asthma action plan. Mothers in Class 2 more frequently had a high education level (79.5% vs. 65.2%, *p* = 0.004). Children in Class 2 more frequently had fully controlled asthma (36.7% vs. 25.9%, *p* = 0.015) and hospitalizations for attacks in the previous 12 months (24.2% vs. 10.7%, *p* = 0.003). LCA can help discover underlying mother profiles and plan targeted educational interventions.

## 1. Introduction

According to international guidelines and strategies, asthma management is essential for achieving and maintaining disease control. In the context of childhood asthma, parents have the primary responsibility of managing their children’s asthma. Thus, they need to be educated regarding the nature of asthma, asthma triggers, medication and devices, self-management techniques, and should receive a written action plan [1].

Current evidence shows that the parental level of asthma-related knowledge may influence asthma management practices [2] and affect children’s disease control [3,4,5,6]. Much of the existing literature studying the impact of parental knowledge on childhood asthma outcomes focuses on mothers as the primary caregivers [5,7,8,9,10,11], suggesting that asthma in children is majorly managed by mothers. Therefore, assessing mothers’ knowledge and practices is essential for improving the management of childhood asthma.

Several questionnaires have been developed for evaluating parental knowledge and practices about childhood asthma [6,12,13,14,15,16,17,18]. However, findings from these studies have highlighted that the construction of reliable self-report asthma knowledge instruments may not be achievable in practice [14]. Specifically, providing reliable scoring systems is a challenging task, mainly due to the lack of a gold standard measurement able to quantify the “true” level of parent knowledge, which is never observed. For this reason, the issue of criterion validity (the correlation between the test and a previously validated criterion taken as representative of the construct) has not sufficiently been addressed in previous studies of asthma knowledge/practice questionnaires [19]. Some attempts to identify subgroups of respondents with “good knowledge” have been based on somewhat arbitrary criteria: subgroups of parents selected by pediatricians [13], subjects receiving education modules [12], mothers with a total score greater than the 50th percentile [11]. On the other side, commonly assessed properties are content and face validity [12,18], internal consistency [15,17], and Test-Retest Reliability [12,17]. Another important aspect to consider is that answers may not always be identified as objectively correct or incorrect, and items may not be equally important [16].

In this study, we aimed to use a model-based approach for identifying different profiles of mothers of asthmatic children based on their knowledge and practices related to childhood asthma, therefore addressing the lack of a gold standard of parent knowledge. In this regard, we hypothesized that Latent Class Analysis (LCA) could be a helpful statistical approach as it assumes that each responder belongs to a latent (unobserved) subgroup, producing expectations about how that responder will answer a set of questions (manifest variables), without explicitly establishing correct or incorrect answers.

## 2. Materials and Methods

### 2.1. Study Design and Population

In this cross-sectional study, mother–child pairs were consecutively recruited during their first consultation at the outpatient clinic of Pediatric Allergology & Pulmonology of CNR-IRIB (Palermo, Italy) between June 2015 and December 2017. The inclusion criteria were: (1) child with a doctor’s diagnosis of asthma according to the “Global Initiative for Asthma” (GINA) guidelines (www.ginasthma.org, last accessed 20 January 2022); (2) child aged 5–16 years; (3) ability to perform spirometry. The exclusion criteria were: (1) mother with poor understanding of the Italian language; (2) child with concomitant chronic diseases (diabetes, congenital and genetic diseases, autoimmune and neuropsychiatric disorders); (3) lack of written informed consent. The study was approved by the local Institutional Ethics Committee (Palermo 1, Italy, No. 10/2014) and was registered on the central registration system ClinicalTrials.gov (ID: NCT02464189). All the participants were informed about all aspects of the research and provided their consent before study entry.

### 2.2. Procedures

At the first consultation, children underwent a clinical examination by well-trained physicians (VM, GF, and SLG) for eligibility assessment. Height (in cm) and weight (in kg) were measured in the standing position without shoes, using a stadiometer (Wunder HR1, Monza, Italy) and an electronic weighing scale (Seca, Hamburg, Germany). Body mass index (BMI, kg/m^2^) standard deviation (SD) scores were derived based on the World Health Organization (WHO) age-specific reference values and were categorized as underweight (below −2 SD), normal (between −2 and 1 SD), and overweight/obese (above 1 SD). Forced expiratory value in 1 s (FEV_1_) and the FEV_1_ and forced vital capacity ratio (FEV_1_/FVC) were measured using a portable spirometer (Pony FX, Cosmed, Rome, Italy) according to standardized guidelines [20] and were expressed as a percentage of predicted values [21]. Asthma severity and control level were assessed according to the GINA guidelines (www.ginasthma.org, last accessed 20 January 2022). The following information related to the child’s asthma was self-reported by the mother: age (years) at symptom onset; age (years) at doctor’s diagnosis; treatment in the previous 12 months, categorized as “no treatment”, “as needed”, or “regular for at least three months” (type of treatment not specified); number of attacks and related emergency department (ED) visits and hospitalizations in the previous 12 months.

Skin prick tests (SPTs) were performed through a Stallerpoint-VC^®^ kit, using a panel of relevant aeroallergens (*Dermatophagoides* mix, *Alternaria alternata*, dog and cat dander, *Parietaria judaica*, grass pollen mix, olive pollen), plus positive (histamine 1%) and negative (saline) controls (Stallergènes Italia Srl., Milan, Italy). Allergens were pricked on the forearm, and reaction sizes were evaluated after 15 min. A positive reaction was defined as a skin response with a wheal ≥3 mm larger than the negative control test [22]. The children were categorized as non-sensitized (no positive reactions), mono-sensitized (one positive reaction), and poly-sensitized (>1 positive reactions). Allergic rhinitis was diagnosed according to “Allergic Rhinitis and its Impact on Asthma” (ARIA) guidelines [23].

The mothers’ BMI was categorized as underweight (below 18.5 kg/m^2^), normal (between 18.5 and 25 kg/m^2^), and overweight/obese (above 25 kg/m^2^). Then, mothers were interviewed through a modified version of the structured SIDRIA questionnaire [24], including questions about sociodemographic characteristics, maternal history of asthma, and current (previous 12 months) environmental exposures. The mothers’ education level was categorized as “primary/lower secondary” and “upper secondary or higher”. We also recorded the number of family members and exposure to environmental tobacco smoke (ETS), pets (dog/cat), and molds in the child’s bedroom.

Finally, mothers underwent a structured interview investigating knowledge and practices about childhood asthma. The questionnaire was composed of 18 questions selected by a panel of expert physicians (VM, AL, GF, and SLG) from a previously developed tool [2,6]. Item selection was aimed to reduce the length of the questionnaire (it was originally composed of 50 questions) and was supported by literature evidence and the professional experience of the researchers. The first nine questions were related to knowledge about asthma etiology, triggering factors, and clinical presentation. The other nine questions concerned practices toward asthma management and compliance to a control plan. The questionnaires were administered by a psychologist (LM), who ensured that all the questions were answered.

### 2.3. Statistical Analyses

LCA was used to discover underlying response patterns, allowing the identification of classes of mothers with similar knowledge and practices. The “poLCA” package [25] of R statistical software (R Foundation for Statistical Computing, Vienna, Austria) was used for estimation. The parameters estimated by LCA are the proportion of mothers in each latent class (prior probabilities of latent class membership) and the response proportions in each latent class (item-response probabilities). Given the observed pattern of item responses, the prior probabilities of latent class membership are updated for each responder (posterior probabilities of latent class membership) and can be used to probabilistically classify the mothers [26].

The mothers’ answers to questions about knowledge and practices were used as manifest variables. Several LCA models were fitted by increasing the number *K* of latent classes from one to five, and the Bayesian Information Criterion (BIC) was used to define the optimal number of classes (the smaller the better) [27]. For the optimal LCA model, the estimated probabilities of positive response were visually displayed, and the classes were labeled accordingly. Posterior probabilities of class membership were computed for each mother, who was assigned to the class associated with the highest probability. Overall classification accuracy was obtained as the mean, over all the mothers, of the maximum posterior probability of latent class membership (assignment probability). Class-specific classification accuracies were derived as the conditional means of the assignment probabilities, which were visually displayed.

Mothers classified with an assignment probability of at least 0.90 were included in subsequent analyses aiming to compare the distribution of mother and child characteristics and the distribution of positive answers among the classes. The Kruskal–Wallis test (quantitative variables) or Fisher’s Exact test (categorical variables) were used for the group comparisons, setting the statistical significance at *p* < 0.05.

## 3. Results

Out of 456 potentially eligible mother–child pairs, 438 consented to participate in the study (Table 1). Mean mother age was 41.8 years. Children were predominantly males (65.5%), and their mean age was 9.1 years. Mean child’s age at doctor diagnosis of asthma was about six years. One-third of the children had intermittent asthma, and 34.2% had fully controlled asthma. About half of the children were treated as needed, while 37.4% had been under regular treatment for at least three months during the last year. Mean FEV_1_ and FEV_1_/FVC (% predicted) were 94.2 and 97.6, respectively. In the previous 12 months, asthma attacks had been experienced by 76.7% of the children, while related ED visits and hospitalizations had been experienced by 37.9% and 19.9% of the children, respectively.

Concerning knowledge items (Table 2), the most frequent positive answers involved allergens as a factor for asthma worsening (83.3%) and repeated cough as an indicator of an asthma attack (80.6%). The least frequent positive answers involved cold air as an asthma-worsening factor (38.6%). Concerning practices (Table 2), the most frequent positive answers involved smoke exposure avoidance (81.7%) and the use of bronchodilators for an asthma attack (78.5%). The least frequent positive answers involved the use of antibiotics for an asthma attack (10.0%).

**Figure 1:**
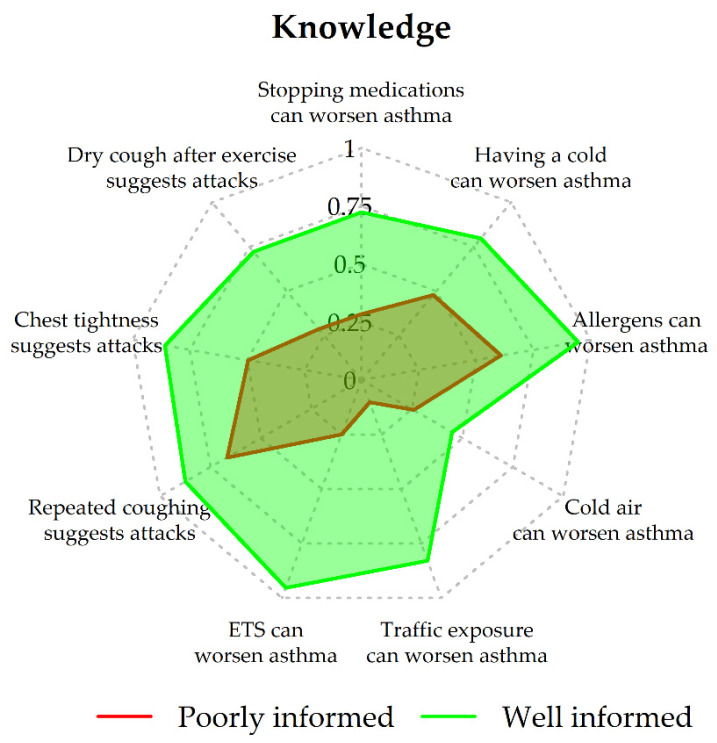
Knowledge: probabilities of positive response by class. ETS: environmental tobacco smoke.

**Figure 2:**
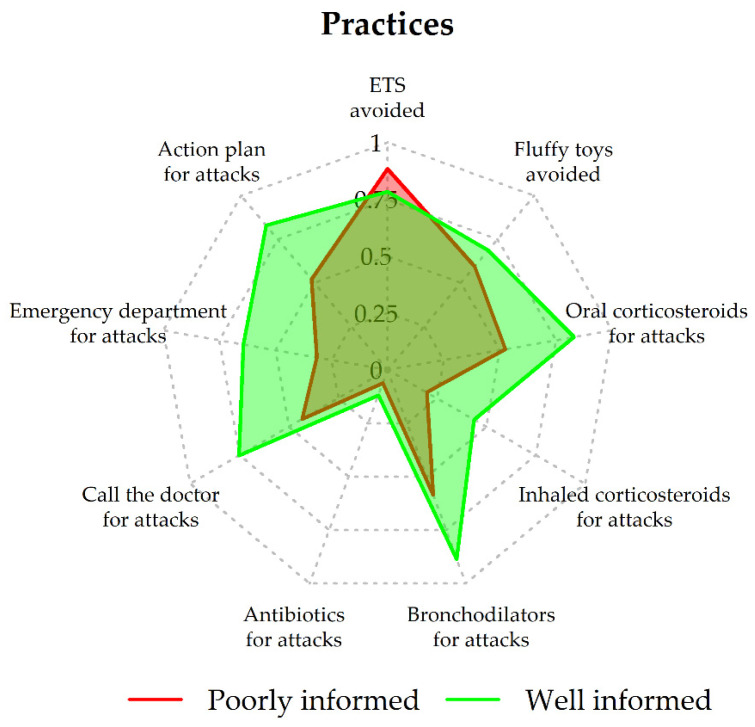
Practices: probabilities of positive response by class. ETS: environmental tobacco smoke.

Based on the BIC, LCA identified two latent classes (1-class BIC, 9366.649; 2-class BIC, 8820.444; 3-class BIC, 8823.062; 4-class BIC, 8842.353; 5-class BIC, 8897.438). According to the estimated item-response probabilities (Figure 1 and Figure 2), the following labels were attributed to the classes: Class 1, “poor knowledge” (prior probability of class membership = 0.33); Class 2, “good knowledge” (prior probability of class membership = 0.67).

Using posterior probabilities, 145 mothers (33%) were assigned to Class 1, while 293 mothers (67%) were assigned to Class 2, consistently with the estimated prior probabilities of class membership (0.33 and 0.67, respectively).

For clinicians interested in assessing it, a posterior probability calculator is freely accessible at https://giovannacilluffo.shinyapps.io/app_polca (last accessed 20 January 2022), in which prior probabilities are dynamically updated while providing one or more answers.

**Figure 3:**
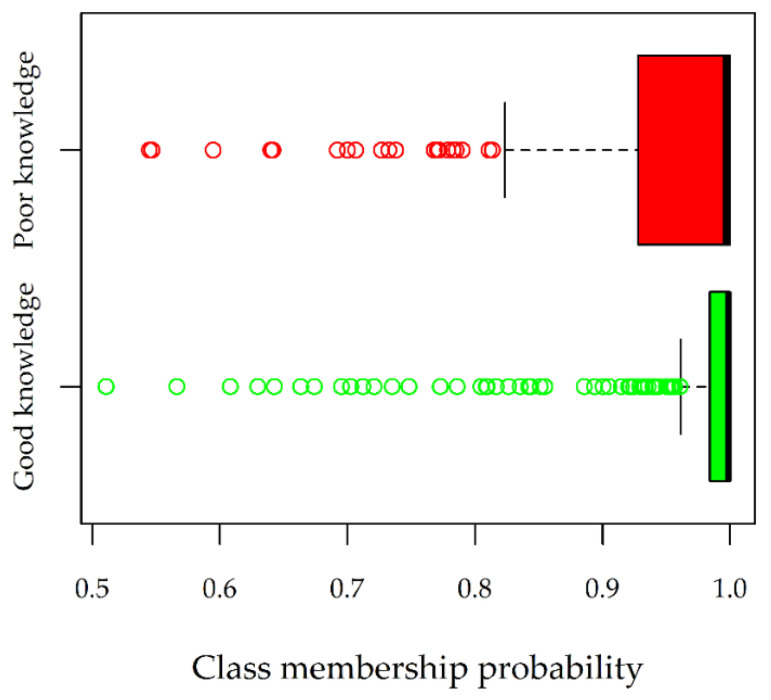
Assignment probability distribution by class. The boxplots represent median values (bold lines next to 1), 25th percentiles (box boundary lines), extreme values (whiskers), and outliers (points).

The mean assignment probability (overall classification accuracy) was 0.96. Classification accuracy was slightly lower in Class 1 (0.94) than in Class 2 (0.97) (Figure 3). The assignment probability was above 0.90 for 376 mother–child pairs (112 in Class 1 and 264 in Class 2), who were included in subsequent analyses.

Mothers in Class 2 were more likely to be aware of asthma-worsening factors and indicators of acute attacks than mothers in Class 1 (Table 2). Whereas mothers in Class 1 were less likely to avoid exposure to fluffy toys, they were more likely to prevent exposure to tobacco smoke than mothers in Class 2 (Table 2). For an asthma attack, mothers in Class 2 were more likely to use oral/inhaled corticosteroids and bronchodilators, while reports of antibiotic use were infrequent and similarly distributed in the two classes. Moreover, mothers in Class 2 were more likely to call the doctor, go to the ED, and follow the asthma action plan (Table 2).

Mothers in Class 2 more frequently had a high education level (upper secondary or higher) than mothers in Class 1 (79.5% vs. 65.2%, *p* = 0.004). For children in Class 1, asthma was less frequently fully controlled (25.9% vs. 36.7%, *p* = 0.015). Mothers in Class 2 more frequently reported hospitalization for asthma attacks in the previous 12 months than mothers in Class 1 did (24.2% vs. 10.7%, *p* = 0.003) (Table 1).

## 4. Discussion

LCA allowed identifying two classes of mothers with similar asthma-related knowledge and practices without explicitly establishing any scoring system based on correct/incorrect answers. The two classes were mainly characterized by different response patterns in the knowledge domain and were therefore labeled as “poor knowledge” (Class 1, 33%) and “good knowledge” (Class 2, 67%). Differences in the response patterns were less marked for the practice domain, with a slightly lower tendency to prevent tobacco smoke exposure observed among mothers in the “good knowledge” class. Higher education levels, fully controlled asthma, and hospitalization for asthma attacks in the previous 12 months were significantly associated with membership in the “good knowledge” class.

Overall, the response patterns observed in the whole sample were consistent with those observed in previous studies. About 60% of mothers believed that stopping taking drugs could worsen their children’s asthma, consistently with previous studies where about 40% of caregivers (predominantly mothers) believed that asthma medications should only be used when children have symptoms (coughing, congestion, or wheezing) [5,28]. Similar percentages were also found in previous studies concerning the knowledge of symptom triggers like flu infections [29], allergens [30], air pollution [31], and cigarette smoke [30] (about 70%, 80%, 60%, and 70%, respectively). Only 40% of mothers recognized cold air as an asthma-worsening factor. This result is consistent with that of a previous study where only 44% of the mothers believed that exercising in cold weather can trigger an asthma attack [10]. Most of the mothers (60–80%) recognized typical indicators of asthma attacks and reported avoiding child exposure to triggering factors, consistently with previous results [11,32]. Finally, concerning the management of asthma attacks, few relevant questions were included in previously developed questionnaires [33]. Similar percentages (about 70%) were observed for the correct practice of using corticosteroids/β2 agonists [34]. Similar percentages were also found concerning the practices of contacting the doctor/going to the ED (about 60% and 50%, respectively) [30]. The practice of following an asthma action plan was not investigated in previous questionnaires.

The percentage of mothers belonging to the “good knowledge” class estimated through the LCA model was 67%. Previous studies identified respondents with good knowledge based on ad hoc criteria, such as 50–60% of questions answered correctly [2,9] or a total score greater than the 50th percentile [11]. In particular, in one study [9], the percentage of “knowledgeable” caregivers (71.6% mothers) was similar (72.4%) to that observed in this study (67%), while it was lower (40–50%) in the other studies [2,11].

Mothers in Class 1 were characterized by substantially lower percentages of positive answers in the knowledge domain. This result would be consistent with the small percentages of parents (about 10–20%) who were able to identify asthma-worsening factors and indicators of asthma attacks in a study population where only 18.31% of parents achieved acceptable scores in the knowledge domain [2]. Moreover, in a study testing an asthma first aid knowledge questionnaire [35] on a sample of highly literate responders (29% parents, 49% pharmacy students, 17% healthcare professionals, 5% teachers), about 90% were able to recognize the main symptoms of an acute attack (cough and difficulty in breathing), as we observed in Class 2. Concerning knowledge of cigarette smoke as an aggravating factor, the results would be in line with the small percentage (about 30%) of mothers answering similarly in a study population where 60% of mothers had poor knowledge scores [11]. Knowledge of traffic exposure as an aggravating factor differed in the two classes, but it appears not to have been investigated in previous questionnaires.

Consistently with previous findings showing that parental level of knowledge may impact asthma management practices [2], mothers in Class 1 were also characterized by lower percentages of positive answers in the practice domain, except for smoke exposure avoidance. In particular, the percentage of mothers who reported always avoiding their child being exposed to tobacco smoke after his/her asthma diagnosis was high in both classes, and it was slightly larger in the “poor knowledge” class. High percentages (>90%) of parents reporting avoiding tobacco smoke exposure/aggravating factors have also been found in the aforementioned study populations that were mainly characterized by poor asthma knowledge scores [2,11]. Indeed, it has been observed that many parents know that ETS might contribute to the development of several illnesses besides asthma, such as colds/upper respiratory tract infections, otitis, and pneumonia [36]. Although almost all (97%) of the mothers in the “good knowledge” class were aware that cigarette smoke can worsen their child’s asthma, a somewhat lower percentage (79%) of them reported always avoiding their child being exposed. This result would be in line with a previous study where 98% of parents agreed that inhaling smoke harms the health of their asthmatic children, while lower percentages of parents reported that smoking in the presence of children is never allowed at home (88%) or in the car (82%) [37].

Concerning the management of acute asthma attacks, correct practices (using oral corticosteroids/short-acting β2 agonists and following the asthma action plan) were more frequently (80–90%) reported by mothers in the “good knowledge” class. This result is in line with that of a previous study [13] where 62/69 (90%) of the parents placed in a “high knowledge” group answered a similar question correctly. Concerning healthcare utilization, practices of calling the doctor and going to the ED were more frequently (60–70%) reported by mothers in the “good knowledge” class as well. This result is consistent with that of a previous study of caregivers of children with high healthcare utilization, where in spite of the knowledge of asthma pathophysiology and preventive and management steps, the caregivers reported being stressed, helpless, and unable to translate knowledge into action at the time of an exacerbation, calling the doctor or seeking care at the ED [30]. In general, whether such practices were correct or incorrect is challenging to establish in the absence of information about the severity of the attacks.

Higher education levels were associated with membership in the “good knowledge” class, consistently with several previous studies [2,7,8,14]. Indeed, mothers with higher education may be more likely to have prior knowledge and more inclined to gather and absorb new information [8].

Membership in the “good knowledge” class was associated with fully controlled asthma (but not with severity and spirometry) in the previous four weeks. As observed by Ho et al. [14], asthma knowledge is a multifaceted phenomenon, affected by complex sociodemographic and contextual factors, and the construction of a self-report asthma knowledge instrument to predict asthma outcomes may be an unfeasible task. Indeed, whereas some studies [3,4,5,6] reported an association between parental asthma knowledge and disease control as in our study, other studies did not [10,12]. This may also be ascribed to the general tendency of parents with poorer knowledge to underestimate the severity of asthma in their children and to report fewer symptoms [10].

Finally, membership in the “good knowledge” class was associated with hospitalization for acute attacks (but not with ED visits) in the previous 12 months. This result would be consistent with previous findings of higher levels of parent asthma knowledge associated with a history of admissions for asthma attacks [38] and, more in general, with more severe forms of the disease (moderate asthma, comorbid allergic rhinitis, frequent physician visits) [2,39]. Indeed, these parents may be more prone to seek more information about the disease [39].

The main strength and novelty of this study is the application of the LCA methodology for identifying different categories of responders. LCA simply hypothesizes that each responder belongs to a latent class with similar response patterns (similar knowledge and practices) and uses observed answers for probabilistically classifying the mothers. The main advantage of this approach is that correct/incorrect answers and most important items do not need to be explicitly established to calculate a total score. Moreover, criterion validity can be assessed in the form of a conventional classification accuracy, which was estimated to be 0.96 in this study.

Classifying mothers allows highlighting the need for targeted educational interventions. For example, interventions for mothers in the “poor knowledge” class may focus on improving knowledge about the importance of keeping on medications during asymptomatic periods, about triggers of attacks, early identification of acute attacks, and their management. Conversely, interventions for mothers in the “good knowledge” class may be focused on improving home management of acute attacks, which may not always require visiting the ED. Reducing barriers to smoking cessation should also be a relevant topic to be investigated by ad hoc trained pediatricians [40].

Educational interventions to improve parental knowledge on asthma have been previously implemented in different settings (i.e., developed and resource-limited countries) and at different levels (i.e., involving both professionals and community members). They included home-based, multicomponent, and multi-trigger interventions (i.e., environmental assessment, guidance on asthma self-management, written and video educational materials, school-based training, programs combining clinical vignettes with interactive discussions, etc.) delivered by certified asthma educators, nurses, or trained practitioners/pediatricians. Most of these interventions resulted in positive outcomes in terms of improvement in parental knowledge, self-efficacy, and asthma attitudes [12,41,42,43,44,45,46,47,48].

Some study limitations should also be acknowledged. The set of 18 items was selected from a previously developed tool [2,6], for which the authors made no explicit mention of repeatability and internal consistency assessment. Moreover, this study was cross-sectional, and participants were recruited at their first consultation at an outpatient clinic. Some asthma outcomes were self-reported and referred to the previous 12 months (acute attacks, ED visits, and hospitalizations), while other outcomes were assessed by the physician at the time of the visit. Therefore, asthma-related knowledge and practices of the mothers might have been influenced by these contextual factors, and a longitudinal study would be needed to assess future outcomes and possible class transitions.

Another limitation is that we only included mothers in the study. However, much of the existing literature studying the impact of parental knowledge on childhood asthma focuses on mothers as the primary caregiver. Mothers have also reported having higher levels of overall knowledge than fathers [29]. Finally, “Don’t know” answers were not included among the response categories, and this might have encouraged guessing. Guessing would determine uncertain probabilities of latent class membership, i.e., close to 0.5. Since such uncertain probabilities were rarely observed (Figure 3), the guessing effect seems to not have been relevant.

## 5. Conclusions

In conclusion, assessing and addressing asthma-related knowledge and practices among mothers of asthmatic children is a critical requirement to achieve improved children health outcomes. In our study, LCA allowed identifying two classes of mothers with similar asthma-related knowledge and practices, labeled as “poor knowledge” and “good knowledge”. Higher education levels, fully controlled asthma, and previous hospitalization for asthma attacks were associated with membership in the “good knowledge” class. LCA can be helpful to discover different profiles of mothers of children with asthma and may assist in the development of targeted educational interventions.

## Figures and Tables

**Table 1:** Distribution of mother and child characteristics in the whole sample and by class membership (mothers classified with an assignment probability of at least 0.90). Data are expressed as means (SD) for quantitative variables, and *n* (%) for categorical variables. Significant *p*-values are in bold.

	Whole Sample (*n* = 438)	Poor Knowledge (*n* = 112)	Good Knowledge (*n* = 264)	*p*-Value
Mother				
Age, years	41.8 (5.7)	41.8 (5.0)	41.8 (5.6)	0.904
Body mass index category				0.780
Underweight	19 (4.3)	5 (4.5)	11 (4.2)	
Normal	261 (59.6)	63 (56.2)	159 (60.2)	
Overweight/Obese	158 (36.1)	44 (39.3)	94 (35.6)	
Upper secondary or higher education	330 (75.3)	73 (65.2)	210 (79.5)	**0.004**
Asthma history	93 (21.2)	19 (17.0)	55 (20.8)	0.478
Environment				
Number of family members	4.0 (0.8)	4.0 (0.9)	4.0 (0.8)	0.595
Environmental tobacco smoke	174 (39.7)	42 (37.5)	104 (39.4)	0.817
Pet in the child’s home (dog/cat)	83 (18.9)	23 (20.5)	49 (18.6)	0.669
Molds in the child’s bedroom	102 (23.3)	29 (25.9)	59 (22.3)	0.506
Child				
Male gender	287 (65.5)	71 (63.4)	169 (64.0)	0.907
Age, years	9.1 (2.6)	9.3 (2.5)	9.1 (2.6)	0.460
First-born	250 (57.1)	61 (54.5)	158 (59.8)	0.361
Body mass index category				0.097
Underweight	6 (1.4)	0 (0.0)	5 (1.9)	
Normal	203 (46.3)	45 (40.2)	128 (48.5)	
Overweight/Obese	229 (52.3)	67 (59.8)	131 (49.6)	
Allergic sensitization				0.323
Non-sensitized	81 (18.5)	25 (22.3)	45 (17.0)	
Mono-sensitized	143 (32.6)	32 (28.6)	93 (35.2)	
Poly-sensitized	214 (48.9)	55 (49.1)	126 (47.7)	
Allergic rhinitis				0.445
No	177 (40.4)	52 (46.4)	105 (39.8)	
Intermittent	99 (22.6)	22 (19.6)	64 (24.2)	
Persistent	162 (37.0)	38 (33.9)	95 (36.0)	
Age at asthma symptom onset, years	4.1 (3.0)	4.4 (3.5)	4.0 (2.9)	0.511
Age at asthma diagnosis, years	5.8 (3.1)	6.0 (3.4)	5.8 (3.0)	0.879
Persistent asthma	292 (66.7)	79 (70.5)	172 (65.2)	0.340
Asthma control status (GINA)				**0.015**
Controlled	150 (34.2)	29 (25.9)	97 (36.7)	
Partly controlled	90 (20.5)	33 (29.5)	45 (17.0)	
Uncontrolled	198 (45.2)	50 (44.6)	122 (46.2)	
Asthma treatment				0.367
No treatment	58 (13.2)	12 (10.7)	37 (14.0)	
As needed	216 (49.3)	63 (56.2)	128 (48.5)	
Regular for at least 3 months	164 (37.4)	37 (33.0)	99 (37.5)	
FEV_1_ % predicted	94.2 (13.5)	95.1 (12.8)	92.8 (13.1)	0.096
FEV_1_/FVC % predicted	97.6 (7.5)	96.4 (7.9)	98.0 (7.5)	0.116
Asthma attacks, previous 12 months				0.450
0	102 (23.3)	68 (25.8)	23 (20.5)	
1	60 (13.7)	38 (14.4)	14 (12.5)	
>1	276 (63.0)	158 (59.8)	75 (67.0)	
ED visits, previous 12 months				0.122
0	272 (62.1)	155 (58.7)	77 (68.8)	
1	104 (23.7)	69 (26.1)	19 (17.0)	
>1	62 (14.2)	40 (15.2)	16 (14.3)	
Hospitalizations, previous 12 months				**0.003**
0	351 (80.1)	200 (75.8)	100 (89.3)	
1	76 (17.4)	56 (21.2)	10 (8.9)	
>1	11 (2.5)	8 (3.0)	2 (1.8)	

**Table 2:** Knowledge and practices: distribution of positive answers in the whole sample and by class membership (mothers classified with an assignment probability of at least 0.90). Data are expressed as *n* (%). Significant *p*-values are in bold.

	Whole Sample (*n* = 438)	Poor Knowledge (*n* = 112)	Good Knowledge (*n* = 264)	*p*-Value
**Knowledge**				
Can stopping taking drugs worsen your child asthma?	253 (57.8)	28 (25.0)	200 (75.8)	**<0.001**
Can having a cold worsen your child asthma?	303 (69.2)	53 (47.3)	213 (80.7)	**<0.001**
Can exposure to allergens worsen your child asthma?	365 (83.3)	59 (52.7)	253 (95.8)	**<0.001**
Can cold air worsen your child asthma?	169 (38.6)	28 (25.0)	122 (46.2)	**<0.001**
Can traffic exposure worsen your child asthma?	257 (58.7)	4 (3.6)	232 (87.9)	**<0.001**
Can tobacco smoke exposure worsen your child asthma?	315 (71.9)	14 (12.5)	256 (97.0)	**<0.001**
Could repeated coughing indicate an asthma attack?	353 (80.6)	71 (63.4)	233 (88.3)	**<0.001**
Could chest tightness indicate an asthma attack?	323 (73.7)	48 (42.9)	233 (88.3)	**<0.001**
Could dry cough after exercise indicate an asthma attack?	253 (57.8)	26 (23.2)	201 (76.1)	**<0.001**
**Practices**				
Have you always avoided your child being exposed to tobacco smoke after his/her asthma diagnosis?	358 (81.7)	102 (91.1)	208 (78.8)	**0.005**
Have you always avoided your child being exposed to fluffy toys after his/her asthma diagnosis?	287 (65.5)	59 (52.7)	180 (68.2)	**0.005**
Will you use oral corticosteroids if your child has an acute asthma attack?	319 (72.8)	52 (46.4)	221 (83.7)	**<0.001**
Will you use inhaled corticosteroids if your child has an acute asthma attack?	157 (35.8)	19 (17.0)	120 (45.5)	**<0.001**
Will you use short-acting β2 agonists if your child has an acute asthma attack?	344 (78.5)	61 (54.5)	240 (90.9)	**<0.001**
Will you use antibiotics if your child has an acute asthma attack?	44 (10.0)	8 (7.1)	34 (12.9)	0.151
Will you call the doctor if your child has an acute asthma attack?	283 (64.6)	46 (41.1)	201 (76.1)	**<0.001**
Will you go to the emergency department if your child has an acute asthma attack?	234 (53.4)	33 (29.5)	177 (67.0)	**<0.001**
Will you follow the asthma action plan if your child has an acute asthma attack?	318 (72.6)	53 (47.3)	226 (85.6)	**<0.001**

## Data Availability

The data that support the findings of this study are available from the corresponding author upon reasonable request.
